# Report of Two Cases: Altered Mental Status and Anisocoria as Presenting Symptoms in Acute Basilar Artery Occlusion

**DOI:** 10.5811/cpcem.48350

**Published:** 2025-09-23

**Authors:** Andrew Ryu, Karizma Chhabra, Thomas George, Elizabeth Kasparov, Mohamed Wali, Christopher C. Lee

**Affiliations:** South Shore University Hospital/Northwell Health, Department of Emergency Medicine, Bay Shore, New York

**Keywords:** anisocoria, basilar artery occlusion, posterior stroke, altered mental status

## Abstract

**Introduction:**

A posterior circulation stroke at the level of the basilar artery can cause ischemia to the brainstem, cerebellum, and occipital lobes. Posterior circulation strokes are notoriously more difficult to clinically diagnose than anterior circulation strokes, with a variety of presenting symptoms including altered mental status, dizziness, vision changes, nausea, and vomiting. Anisocoria has been reported to occur in rare cases.

**Case Report:**

We present two cases where patients had an acute episode of altered mental status with a key exam finding of anisocoria, or unequal pupil sizes. The combination of anisocoria and acute mental status decline are classically associated with traumatic brain injury, increased intracranial pressure, or both. In each of the two cases presented, acute basilar artery occlusion was seen on computed tomography with angiography.

**Conclusion:**

When presented with acute decline in mental status and anisocoria, early clinical suspicion of an acute basilar artery occlusion is crucial in diagnosing and managing these patients with debilitating acute posterior stroke. Time-sensitive interventions such as thrombolytics and mechanical thrombectomy can be lifesaving.

## INTRODUCTION

The posterior circulation of the brain refers to the vertebrobasilar vascular system, beginning with the vertebral arteries coming off the subclavian arteries that join to form the basilar artery. A posterior circulation stroke at the level of the basilar artery can cause ischemia to the brainstem, cerebellum, and occipital lobes, causing a wide range of symptoms including an acute change in mental status as well as vestibular and ocular symptoms.^1^ Anisocoria has been reported to occur in rare cases of posterior circulation strokes.^2^–^4^ Anisocoria is defined as unequal size of the pupils. Mydriasis is primarily mediated by the sympathetic neuronal input, while miosis is mediated by parasympathetic input. Anisocoria is caused by disruption and mismatch of these neuronal inputs. Possible etiologies include physiologic anisocoria in up to 20% of the population, increased intracranial pressure and, in rare instances, posterior circulation strokes.^5^ We describe two cases of acute basilar artery occlusion in patients who had acute episodes of altered mental status with anisocoria on physical exam.

## CASE REPORT

### Patient 1

A 61-year-old female with past medical history of acute ischemic stroke and left atrial appendage thrombus treated with apixaban presented to the emergency department (ED) after a mechanical fall. The patient’s son found her on the ground with baseline mental status after a fall from standing resulting in head trauma. Computed tomography (CT) of the head and cervical spine did not show any acute findings. The patient was admitted due to elevated troponin markers per cardiologist’s recommendations.

**Image 1:**
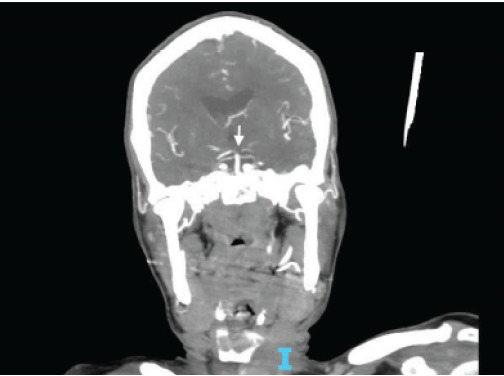
Computed tomography angiography coronal view (Patient 1) with arrow pointing to proximal basilary artery occlusion with multifocal occlusions of the left posterior cerebral artery.

During her stay overnight, the rapid response team was activated for an acute mental status change, with last known well three hours prior. The patient was minimally responsive to pain with snoring respirations and a National Institute of Health Stroke Scale score of 22. On physical exam, the right pupil was 1 mm in diameter and reactive to light, and the left pupil was 4 mm and nonreactive. An emergent stroke workup was initiated, and CT angiography (CTA) of her head showed multifocal occlusions of the left posterior cerebral artery (PCA) (Image 1). The stroke neurologist communicated concerns for proximal basilar artery occlusion, and the patient was taken for emergent mechanical thrombectomy. Basilar tip occlusion was found. Thrombolysis in cerebral infarction grade 3 reperfusion was achieved after first pass, and the patient was admitted to the neurointensive care unit. The patient expired during her stay, which was complicated by development of aspiration pneumonia.

### Patient 2

An 80-year-old female with past medical history of atrial fibrillation treated with rivaroxaban presented to the ED with syncope. The patient was in the kitchen when her family heard her fall, and she was minimally responsive afterward with blue lips and trouble breathing. On arrival, the patient was minimally responsive to pain and agitated. She was intubated for airway protection. On physical exam, her right pupil was 6 mm and nonreactive to light, and her left pupil was 4 mm and reactive. The patient had a trauma workup with CT head and cervical spine without contrast, CTA head and neck, CT chest, abdomen, and pelvis with intravenous (IV) contrast, which showed no acute traumatic findings.

*CPC-EM Capsule*

What do we already know about this clinical entity?

*An acute basilar artery occlusion can cause ischemia to the brainstem, cerebellum, and occipital lobes, causing a wide range of symptoms including ocular symptoms*.

What makes this presentation of disease reportable?

*No previous studies have described the prevalence of anisocoria and altered mental status as presenting symptoms in acute basilar artery occlusion*.^11^

What is the major learning point?

*Prompt consultation with the stroke radiologist or stroke neurologist may be warranted to communicate concerns for a possible acute posterior stroke*.

How might this improve emergency medicine practice?

*By creating more awareness, these stroke patients can be identified more quickly and potentially be candidates for thrombolytics or mechanical thrombectomy*.

**Image 2:**
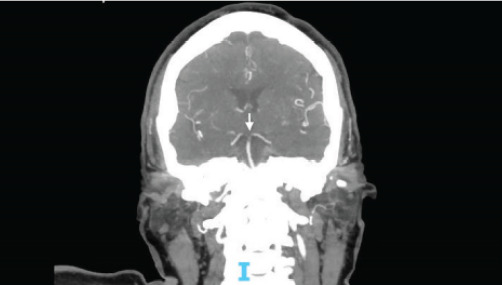
Computed tomography angiography coronal view (Patient 2) with arrow pointing to the site of basilar artery occlusion with bilateral posterior cerebral artery involvement.

The patient was admitted to the medical intensive care unit for further management. Repeat CT head without contrast was done a day after admission due to a decrease in responsiveness, showing acute infarcts in the bilateral cerebellar hemispheres and cerebellar vermis with mass effect on the fourth ventricle and rostral hydrocephalus. The initial CTA head result was addended at this time to show diminished flow in the distal basilar artery extending into the origins of the bilateral PCAs compatible with intraluminal thrombus (Image 2). Neurology and neurosurgery were consulted, and the patient was deemed not a candidate for advanced therapies or surgeries, given devastating neurological injury and poor prognosis. The decision was made with family to withdraw care, and the patient expired on the third day of admission.

## DISCUSSION

Stroke is a critical condition in which time-sensitive interventions such as fibrinolytics or endovascular thrombectomy can be lifesaving for the patient. This case report shows that an acute basilar artery occlusion should be considered as a possible etiology for patients who present with acute change in mental status and anisocoria, even if there is a high clinical suspicion for acute traumatic brain injury as in the case of Patient 2. A stroke workup includes CTA of the brain and neck and CT perfusion, which are often read emergently by the stroke radiologist to aid in the time-sensitive management of acute ischemic strokes. In the case of Patient 2, a trauma workup was pursued instead of a stroke workup to rule out traumatic intracranial hemorrhage. In the report for the CTA head ordered as part of the trauma workup, the basilar artery occlusion extending into the bilateral posterior cerebellar arteries was initially missed and then later included in the addendum.

Posterior circulation occlusions are known to be difficult to localize on CTA due to the anatomical and functional complexity of the posterior vasculature with high frequency of anatomical variants, such as hypoplastic arteries and congenital anomalies.^6^–^7^ Furthermore, since a stroke workup was not pursued in this case, a CT perfusion study was not ordered, which can greatly improve the diagnostic accuracy in acute posterior circulation strokes.^8^ In retrospect, it is not possible to know whether an emergent stroke workup may have led to earlier diagnosis of a basilar artery occlusion on the CTA. Posterior circulation strokes are notoriously more difficult to clinically diagnose than anterior circulation strokes, with a variety of presenting symptoms including altered mental status, dizziness, vision changes, nausea, and vomiting. Altered mental status has been reported to have been present in up to 25% of missed stroke cases.^6^, ^9^–^10^ This case demonstrates that prompt consultation with the stroke radiologist or stroke neurologist may be warranted to communicate concerns for a possible acute posterior stroke in case presentations such as these.

## CONCLUSION

While anisocoria is a known symptom of posterior circulation strokes, we did not find any previous studies that described the prevalence of anisocoria and altered mental status as presenting symptoms in acute basilar artery occlusion.^11^ By raising awareness of this symptomatology, these stroke patients can be identified more quickly and accurately in time-sensitive, emergent stroke evaluations and potentially be candidates for life-saving thrombolytics or mechanical thrombectomy.
